# Adverse Lifestyle Leads to an Annual Excess of 2 Million Deaths in China

**DOI:** 10.1371/journal.pone.0089650

**Published:** 2014-02-26

**Authors:** G. Neil Thomas, Man Ping Wang, Sai Yin Ho, Kwok Hang Mak, Kar Keung Cheng, Tai Hing Lam

**Affiliations:** 1 Public Health, Epidemiology, and Biostatistics, University of Birmingham, Birmingham, United Kingdom; 2 Institute of Public Health, Social and Preventive Medicine, Mannheim Medical Faculty, University of Heidelberg, Mannheim, Germany; 3 School of Nursing, University of Hong Kong, Hong Kong SAR, China; 4 School of Public Health, University of Hong Kong, Hong Kong SAR, China; 5 Department of Health, Hong Kong SAR Government, Hong Kong SAR, China; Iran University of Medical Sciences, Iran (Republic of Islamic)

## Abstract

**Background:**

Adverse lifestyle factors have been associated with increased mortality, but data are lacking on their combined effect in developing populations, which we address in the present study.

**Methods:**

In a death registry-based, case-control study among Hong Kong Chinese aged 30+y, proxy-reported lifestyle factors 10 y ago were collected for 21,363 cases (81% of all deaths) and 12,048 living controls. Risks associated with poor diet, inactivity, heavy alcohol intake, and smoking for all-cause and cause-specific mortality, adjusting for potential confounders, were determined, and excess deaths for the Chinese population were calculated.

**Results:**

Adjusted odds ratios for all-cause mortality were 1.15 (95% CI 1.09, 1.23), 1.34 (1.27, 1.43), 1.36 (1.21, 1.52), and 1.58 (1.46, 1.70) for poor diet, inactivity, heavy alcohol intake and smoking, respectively. Increasing numbers of adverse lifestyle factors were associated with a dose-dependent increase in adjusted odds ratios of 1.30 (1.20, 1.40), 1.67 (1.54, 1.81), 2.32 (2.08, 2.60), and 3.85 (3.12, 4.75) for 1, 2, 3, and 4 risk factors relative to those with none. The population attributable fraction for all-cause, all-CVD and all-cancer mortality were 26.6%, 15.0%, and 32.1%, resulting in an excess of 2,017,541; 489,884; and 607,517 deaths annually, respectively. Although smoking was associated with the greatest excess loss of life (867,530), heavy drinking (680,466), and physical inactivity (678,317) were similarly important.

**Conclusion:**

Adverse lifestyle factors contribute to one quarter of all deaths in China. Improving lifestyle practices, particularly focussing on moderating alcohol intake and increasing activity, and smoking cessation are critical to reducing the lifestyle-associated health burden.

## Introduction

The economic transition to a developed economy has far sweeping implications on individuals. Mechanisation both at work and at home with improved transportation have greatly reduced the need for physical activity [1]. Likewise, mass production has increased the availability of processed, calorie-dense, salty foods [1]. These changes have been widely implicated in the increasing burden of chronic disease worldwide [1], [2], [3]. Apart from physical inactivity and poor diet, smoking and excessive alcohol intake are also major lifestyle risk factors of mortality [2], [4].

Lifestyle factors predisposing to diseases are multi-factorial and inter-related, and do not exist alone in an individual, yet the data describing the combined effects on all-cause and disease-specific mortality are limited, even in developed Western populations [2], [5], [6], [7], [8], [9], . Many of those have also included obesity as a lifestyle measure [10], [11], [12], [13], [14], [15], yet this is a composite measure of lifestyle rather than a lifestyle measure specifically. Only a few studies in Asian populations have attempted to address this issue [14], [15]. The Japan Collaborative Cohort described in two separate reports a dose-dependent increase in risk of death with an increasing number of adverse lifestyle parameters, although the reference group included up to two of six potentially adverse risk factors and thus probably underestimated the risk [15], [16]. In Singapore, healthy lifestyle factors were also beneficial in reducing cardiovascular mortality in older individuals (45 y and over), although included BMI as a lifestyle factor [17]. In data describing Taiwanese diabetics, adverse lifestyle factors were associated with an increase in all-cause and cardiovascular, but not cancer, mortality [18]. In two studies from Korea males, harmful lifestyle factors, both including BMI, were used to construct a risk score, with those with a higher score being at increased risk of all-cause mortality [19], [20]. However, as with the Japanese study, both included harmful factors in the reference group [19], [20]. In developing countries, only a single study of older married, non-smoking, non-drinking women from China has tried to address this issue [14]. The investigators were unable to investigate the impact of smoking or excessive alcohol consumption due to the low prevalence in that population [14].

We aimed to investigate for the first time the impact of the combined effect of adverse lifestyle factors on all-cause and disease-specific mortality in Hong Kong, the most economically-advanced city in China and is illustrative of what is expected to occur in China as the rapid economic development continues unabated. The risk estimates were then used to extrapolate the associated burden to the remainder of China, the world’s most populous nation.

## Methods

### Ethical Statement

Consistent with local practice for questionnaire surveys, written consent by subjects was not required and participation constituted consent. The voluntary basis of the study was clearly stated on the questionnaire and explained by the interviewers. The consent process was approved by the Ethics Committee of the Faculty of Medicine, University of Hong Kong.

### Study Design

The LIMOR (LIfestyle and MORtality) study is a large population-based, unmatched, case-control study of 81% of all adult deaths among ethnic Chinese residents in Hong Kong between mid-December 1997 and mid-January 1999. The details of LIMOR study have been reported elsewhere [3], [4]. All lifestyle factor exposures were based on usual habits 10 years prior to data collection for both cases and controls. Relatives who were waiting for the compulsory registration of deaths (cases) at any of the 4 death registries were invited to complete proxy information on demographic characteristics (age, sex, place of birth, education, housing and job type), and health behaviours (smoking, use of alcohol, leisure exercise, and dietary habits) 10 years before the death of the case using a standardised questionnaire. The same information was also obtained for a living third person (controls), other than the informant, either the decedents’ spouse or a person, preferably of a similar age, with whose habits 10 years ago the informant was most familiar. When the informant was the spouse an eligible control was seldom obtained, therefore there were more cases than controls, especially in the older age-groups, and our method tended to select female controls, as wives generally survive their husbands.

The informant was usually an adult child of the decedent, often living in close, multi-generational Chinese families. Proxy reports were chosen first for expediency, second because of the collectivist values of Chinese society, and third because proxies have been shown to be capable of providing reliable answers to simple questions such as those in the present study [21], particularly in Hong Kong [22]. In addition, proxy reports of lifestyle parameters are potentially preferable because of socio-cultural pressures to present oneself as having a healthy lifestyle [23]. Proxy reports have been validated with standard measures [21], [22]. Consumption or intake was generally recorded with multiple categories describing the frequency of intake, for instance to calculate amount of alcohol intake in grammes or for instance, fruit consumption was specified as ‘not at all’, ‘monthly less than once’, ‘monthly 1–3 times’, ‘weekly 1–3 times’, and ‘weekly at least four times’, for the purpose of the present study. These items were collapsed to binary parameters for the analyses.

Subjects who were home-bound (n = 845) or seriously ill (n = 1,792) 10 years before the collection of the data were excluded. A total of 33,411 individuals (21,363 cases and 12,048 controls) aged 30 or above were included in the data analysis. Repeat interviews were conducted to assess reliability by telephone, which is almost universally accessible in Hong Kong, on a random sample of 235 cases and 106 proxy controls, on average three weeks after the initial interview. The levels of agreement was high for the cases and controls, respectively, at 92 and 97% for fruit intake, 66 and 73% for drinking frequency, 73 and 64% for leisure time physical activity, and 92 and 92% for ever smoking status. The level of agreement should be even higher in the current study due to the aggregation of the multiple consumption levels. Causes of death based on International Classification of Diseases-9th revision (ICD-9) were provided by the Department of Health.

### Measurement

Smoking was categorized into current smokers and never smokers (reference group), with ex-smokers excluded. Alcohol consumption was categorized as heavy drinking if ≥27.4 g ethanol/occasion in males or ≥13.7 g ethanol/occasion in females or non-heavy drinking (reference group), with ex-drinkers excluded. Leisure-time physical activity was defined as any form of physical exercise or activity outside the formal work setting and culturally specific examples were provided such as traditional Chinese tai chi or morning exercises commonly practiced by many Hong Kong adults and elderly individuals. Leisure-time physical inactivity was classified as inactivity, namely partaking in physical activity less than once per week and was compared to those who were physically active two or more times per week (reference group). Fruit consumption was used as the proxy for a good diet, and was based on frequency of consumption, which could be up to 21 times per week (once each for breakfast, lunch and dinner). A poor diet was categorized as consumption of fruit less than 7 times per week and was compared to those consuming fruit ≥7 times per week (reference group). Fruit, rather than fruit and vegetables, was chosen due to the very high consumption of vegetables, which would have diminished the ability to discriminate dietary differences between individuals. A total of 5,231 ex-smokers and ex-alcohol consumers were excluded from the analyses, due to potential issues of reverse causality, leaving 33,411 (21,363 cases and 12,048 controls) for the analyses.

### Statistical Analysis

Logistic regression with alive or dead as the dependent variable was used to calculate the adjusted mortality odds ratios for each lifestyle parameter adjusting for sex, age (categorised into 5 year age groups), education (no/primary, secondary, tertiary or higher), place of birth, housing (public housing, hut/shared, self owned, quarter/others), and job type (sedentary, light, moderate, heavy, none). A significance level of 0.05 was applied for all analyses. Model fit was satisfactory with a Nagelkerke R^2^ of 0.21–0.28.

Population attributable fraction for all-cause and cause-specific mortality were calculated for the individual lifestyle factors using Levin’s formula (PAF = (E · (OR-1))/(E · (OR-1) +1)*100; where E is the prevalence of the lifestyle factor in the population [24]. Likewise, for each of the four combined levels of adverse lifestyle factors the approach outlined by Miettinen was employed, (PAF = (E1 · (OR-1)+E2 · (OR2-1)+E3 · (OR3-1)+ E4 · (OR4-1))/(E1 · (OR1-1)+E2 · (OR2-1)+E3 · (OR3-1)+ E4 · (OR4-1) +1)*100); where En is the prevalence of the exposure to the lifestyle factor in that risk strata and ORn is the odds ratio for that risk strata. The combined PAF for all lifestyle factor categories is simply the sum of the PAF for each exposure category [24]. Given the large differences in smoking and alcohol consumption between the males and females, these were calculated for each gender and summed to achieve the total. Total avoidable death was calculated based on the total number of all-cause, all-CVD, all-cancer deaths in people aged 30 or above in China and the combined risk was estimated based on the mutually adjusted results thus taking account the share effects of individual risk factors.

The prevalence data for China was collated from a number of nationally-representative sources to enable matching of the lifestyle factor definitions supporting the validity of the prevalence assumptions. The prevalence of physical activity was derived from the 2002 China National Nutrition and Health Survey [25]. This estimate will be conservative given it investigated activity in 21,834 subjects aged 18–59 years who were employed, of whom 11.8% (15.8% male, 7.3% female) were sedentary and a further 13.4% (12.5% male, 14.4% female) had low levels of activity giving 25.2% (28.3% male, 21.7% female) with of inactive individuals. The prevalence of tobacco use was based on data collated by the World Health Organization, in which 67% of males and only 1% of females were current smokers, an average of 36.4% of the adult Chinese population [26]. The China Health and Nutrition Survey, a nationally representative population-based study of 9 provinces in China, indicated that 42.0% of male drinkers drank ≥25 g alcohol daily intake, whereas 25.3% of female drinkers consumed ≥15 g daily [27]. The 2008 Census of the Chinese population indicated there were approximately 1.3 billion people living in China maintaining its status as the most populous nation, of whom 816,251,793 were aged 30 or more (49.7% male). The overall mortality rate was 7.03 deaths/1,000 with 9,418,266 deaths recorded, of which 7,868,032 (83.5%) were in those aged 30 or above. The 2008 cause-specific mortality data were derived from the Disease Surveillance Point System of the Chinese Centres Disease Control, which is a nationally representative sample including 161 urban and rural sites throughout all 31 provinces, covering a population of 71 million.

## Results

Lifestyle data were available for 33,411 individuals, aged 30 and above who were not home-bound or seriously ill or ex-smokers or ex-drinkers, 10 years before the collection of the data. Table 1 describes the demographic characteristics for both the cases (n = 21,363) and controls (n = 12,048). The odds ratios for the association between the lifestyle parameters and all-cause and cause-specific mortality after adjusting for sex, age (5 yrs group), education (no/primary, secondary, tertiary or higher), place of birth, housing (public housing, hut/shared, self owned, quarter/others), and job type (sedentary, light, moderate, heavy, none) are shown in Table 2. Each of the adverse lifestyle parameters was significantly associated with a higher odds of all-cause mortality, with the strongest effect observed in current smokers (OR 1.58, 95% CI: 1.46, 1.70). The adverse lifestyle factors also had significantly higher odds of all-cancer and all-respiratory deaths. All were also associated with all-CVD deaths, except alcohol which overall showed a higher non-significant odds of 1.14 (95% CI 0.97, 1.33), although this reached significance in the male heavy drinkers (OR 1.20 (1.00, 1.44), p<0.05) (data not shown in tables). Likewise, when we examined increasing numbers of adverse lifestyles, a clear dose-dependent association was observed (Table 3), with a higher odds of 1.33 (95% CI 1.29, 1.37) for all-cause mortality for each additional adverse lifestyle factor. Similar significantly higher odds for each increase in lifestyle parameter, ranging from 1.22 to 1.53, was observed for all major causes of death.

**Table 1:** Background characteristics of the 33,411 cases and controls in Hong Kong^a^.

	Case		Control		All	
	n	%	n	%	N	%
Males	11,273	52.8	3,232	26.8	14,505	43.4
Age (Mean, SD)	71.9	±14.2	67.9	±10.9	70.4	±13.2
Place of birth						
Hong Kong	3,919	18.4	1,844	15.3	5,763	17.3
Guangdong (China)	14,853	69.7	8,800	73.2	23,653	71.0
Other places	2,539	11.9	1,384	11.5	3,923	11.8
Housing type						
Public estate	9,700	48.9	5,860	48.9	15,560	46.7
Self-owned	8,037	41.3	4,955	41.3	12,992	39.0
Hurt/shared	2,199	5.5	659	5.5	2,858	8.6
Quarter/others	1,405	4.3	521	4.3	1,926	5.8
Education						
None	8,624	40.9	5,057	42.4	13,681	41.4
Primary	7,828	37.1	4,725	39.7	12,553	38.0
Secondary	3,845	18.2	1,837	15.4	5,682	17.2
Tertiary	803	3.8	299	2.5	1,102	3.3
Job type history						
Sedentary	2,660	12.6	1,156	9.7	3,816	11.6
Light/medium	10,937	51.8	5,609	46.9	16,536	50.1
Heavy	3,179	15.1	1,425	11.9	4,604	13.9
None	4,312	20.5	3,768	31.5	8,080	24.5
Heavy drinker	2,276	10.7	589	4.9	2,865	8.6
Ever smoked	8,184	38.4	2,454	20.4	10,638	31.9
Exercise <4 times/month	15,192	73.2	7,656	64.8	22,848	70.2
Fruit <7 times/wk	6,545	36.6	2,875	28.5	9,420	33.7
Combined risk factors						
0	2,602	14.8	2,394	24.1	4,996	18.2
1	6,188	35.3	4,294	43.2	10,482	38.1
2	5,377	30.7	2,447	24.2	7,824	28.5
3	2,592	14.8	690	6.9	3,282	11.9
4	786	4.5	123	1.2	909	3.3

a Excluded ex-smokers and ex-drinkers.

**Table 2:** Adjusted odds ratios for individual risk factors and mortality^a^.

Cause of death	N of death	Diet	PA	Alcohol	Smoking
All-cause	21,363	1.15 (1.09, 1.23)***	1.34 (1.27, 1.43)***	1.36 (1.21, 1.52)***	1.58 (1.46, 1.70)***
All-CVD	5,725	1.09 (1.01, 1.19)*	1.25 (1.24, 1.47)***	1.14 (0.97, 1.33)	1.32 (1.19, 1.45)***
IHD	2235	1.17 (1.03, 1.31)*	1.40 (1.23, 1.58)***	0.85 (0.67, 1.07)	1.28 (1.12, 1.48)***
All-respiratory	3,581	1.25 (1.12, 1.38)***	1.67 (1.49, 1.87)***	1.24 (1.04, 1.49)*	2.01 (1.78, 2.27)***
Cardio-pulmonary	9,306	1.15 (1.07, 1.24)***	1.44 (1.34, 1.56)***	1.18 (1.02, 1.56)*	1.55 (1.42, 1.70)***
All-stroke	2,312	0.98 (0.87, 1.06)	1.29 (1.15, 1.45)***	1.40 (1.14, 1.72)**	1.32 (1.14, 1.51)***
Haemorrhagic	851	1.02 (0.86, 1.22)	1.15 (0.96, 1.37)	1.59 (1.20, 2.12)***	1.42 (1.15, 1.75)**
Non-haemorrhagic	1,461	0.95 (0.82, 1.10)	1.39 (1.20, 1.60)***	1.24 (0.95, 1.62)	1.24 (1.04, 1.48)*
Ischaemic	162	0.75 (0.50, 1.13)	1.30 (0.88, 1.91)	1.39 (0.67, 2.91)	0.74 (0.45, 1.24)
Others	1,299	0.98 (0.84, 1.14)	1.39 (1.19, 1.62)***	1.23 (0.93, 1.62)	1.31 (1.09, 1.57)**
All Cancer	8,066	1.10 (1.02, 1.19)*	1.24 (1.15, 1.34)***	1.47 (1.29, 1.68)***	1.93 (1.77, 2.11)***
Lung cancer	2,318	1.11 (0.99, 1.24)	1.23 (1.09, 1.40)**	1.27 (1.06, 1.51)**	4.69 (4.11, 5.34)***
Liver cancer	999	0.93 (0.79, 1.10)	1.24 (1.04, 1.48)*	1.76 (1.39, 2.23)***	1.24 (1.03, 1.49)*
Colorectal cancer	977	1.16 (0.99, 1.34)	1.21 (1.03, 1.43)*	1.40 (1.06, 1.86)*	0.89 (0.73, 1.09)
Stomach cancer	495	0.99 (0.80, 1.24)	1.27 (1.01, 1.60)*	1.20 (0.83, 1.73)	1.19 (0.93, 1.54)
Oesophagus cancer	258	1·39 (1.03, 1.88)*	1.58 (1.07, 2.32)*	5.81 (4.19, 8.06)***	3.07 (2.07, 4.53)***
Breast cancer	310	0.82 (0.60, 1.13)	1.21 (0.91, 1.62)	0.94 (0.39, 2.25)	1.28 (0.81, 2.03)
Prostate cancer	102	0.66 (0.40, 1.09)	1.28 (0.77, 2.11)	1.91 (1.04, 3.51)*	1.21 (0.74, 1.96)
Injury and poisoning	817	1.46 (1.21, 1.75)***	1.09 (0.89, 1.33)	1.23 (0.89, 1.70)	1.16 (0.94, 1.45)

a Adjusting for sex, age (5 yrs group), education (no/primary, secondary, tertiary or higher), place of birth, housing (public housing, hut/shared, self owned, quarter/others), job (sedentary, light, moderate, heavy, none) and the other variables in the table. Reference odds ratio is “1” for fruit intake 7 times or above per week, physical activities 2 times or above per week, non-heavy drinking and never-smoking.

*P<0.05, **P<0.01, ***P<0.001.

**Table 3:** Adjusted odds ratios for combined risk factors and mortality^a^.

	Risk factors						
	0	1	2	3	4	Each increase	P for sex interaction
Subject (N)	4,996	10,482	7,824	3,282	909		
All-cause	1	1.30 (1.20, 1.40)***	1.67 (1.54, 1.81)***	2.32 (2.08, 2.60)***	3.85 (3.12, 4.75)***	1.33 (1.29, 1.37)***	0.03*
All-CVD	1	1.29 (1.16, 1.44)***	1.49 (1.32, 1.67)***	1.94 (1.66, 2.26)***	2.41 (1.81, 3.20)***	1.23 (1.18, 1.28)***	0.81
IHD	1	1.33 (1.13, 1.56)**	1.59 (1.34, 1.89)***	2.01 (1.61, 2.50)***	1.64 (1.08, 2.51)*	1.22 (1.15, 1.30)***	0.50
All-respiratory	1	1.45 (1.24, 1.68)***	2.37 (2.03, 2.77)***	3.45 (2.84, 4.19)***	5.29 (3.87, 7.24)***	1.53 (1.45, 1.62)***	0.07
Cardio-pulmonary	1	1.34 (1.22, 1.48)***	1.76 (1.59, 1.96)***	2.39 (2.08, 2.75)***	3.33 (2.59, 4.27)***	1.34 (1.29, 1.39)***	0.27
All-stroke	1	1.28 (1.10, 1.49)**	1.31 (1.11, 1.54)**	1.82 (1.46, 2.26)***	2.77 (1.93, 3.40)***	1.20 (1.13, 1.27)***	0.05*
Haemorrhagic	1	1.32 (1.04, 1.68)*	1.41 (1.09, 1.82)**	1.69 (1.22, 2.34)**	3.42 (2.13, 5.49)***	1.22 (1.12, 1.33)***	0.50
Non-haemorrhagic		1.26 (1.05, 1.51)*	1.24 (1.02, 1.52)*	1.89 (1.45, 2.45)***	2.19 (1.38, 3.47)***	1.18 (1.10, 1.27)***	0.08
Ischaemic	1	1.19 (0.75, 1.89)	0.98 (0.58, 1.66)	0.73 (0.31, 1.65)	1.58 (0.44, 5.61)	0.97 (0.79, 1.18)	0.18
Others	1	1.28 (1.05, 1.55)*	1.29 (1.05, 1.59)*	2.04 (1.56, 2.68)***	2.30 (1.42, 3.73)**	1.21 (1.12, 1.31)***	0.15
All Cancer	1	1.24 (1.12, 1.36)***	1.60 (1.45, 1.78)***	2.51 (2.20, 2.88)***	4.27 (3.27, 5.50)***	1.37 (1.32, 1.42)***	0.07
Lung cancer	1	1.29 (1.08, 1.65)**	2.40 (2.00, 2.87)***	4.60 (3.75, 5.64)***	6.82 (5.05, 9.21)***	1.71 (1.62, 1.81)***	0.06
Liver cancer	1	1.08 (0.85, 1.36)	1.26 (0.99, 1.60)	1.46 (1.10, 1.95)*	2.91 (1.94, 4.35)***	1.21 (1.12, 1.31)***	0.41
Colorectal cancer	1	1.17 (0.95, 1.43)	1.20 (0.96, 1.50)	1.25 (0.92, 1.70)	2.57 (1.64, 4.04)***	1.13 (1.04, 1.22)**	0.70
Stomach cancer	1	1.27 (0.93, 1.72)	1.24 (0.89, 1.73)	1.59 (1.07, 2.36)*	2.15 (1.17, 3.93)*	1.15 (1.03, 1.28)*	0.97
Oesophagus cancer	1	1.35 (0.68, 2.70)	2.82 (1.25, 5.29)**	7.36 (3.73, 14.52)***	30.89 (15.09, 63.21)***	2.59 (2.22, 3.02)***	0.68
Breast cancer	1	1.48 (1.06, 2.07)*	1.00 (0.65, 1.52)	1.96 (0.97, 2.97)	N/A	1.05 (0.89, 1.24)	-
Prostate cancer	1	1.70 (0.78, 3.68)	1.20 (0.53, 2.69)	2.28 (1.00, 5.21)*	1.46 (0.37, 5.67)	1.14 (0.92, 1.41)	-
Injury and poisoning	1	1.17 (0.89, 1.54)	1.62 (1.22, 2.15)**	1.52 (1.07, 2.17)*	3.02 (1.80, 5.06)***	1.24 (1.13, 1.36)***	0.98

a Adjusting for sex, age (5 yrs group), education (no/primary, secondary, tertiary or higher), place of birth, housing (public housing, hut/shared, self owned, quarter/others) and job (sedentary, light, moderate, heavy, none).

*P<0.05, **P<0.01, ***P<0.001.

After establishing the association between the adverse lifestyle factors and mortality, we then assessed the proportion of events that were associated with those measures (Table 4). The adverse lifestyle factors accounted for a third of all-cause deaths in males, and almost 50% for all-cancer deaths. These estimates do not include indirect deaths such as from passive smoking or traffic accidents. Smoking was the strongest component of those all-cause and all-cancer excess deaths, accounting for 18.6 and 32.8% of those deaths. This translated into 867,530 and 407,494 deaths for all-cause and all-cancer deaths, respectively. The strongest determinant of all-CVD deaths was physical inactivity which accounted for 9.2% of deaths and increased mortality by 257,530 deaths. In total, 2,017,541 or 25.6% of all deaths in China were associated with these lifestyle parameters, of which 489,884 (15.0%) were of CVD origin, and 607,517 (32.1%) were cancers.

**Table 4:** All-cause, all-CVD and all-cancer Population Attributable Risk (%) and total avoidable lives lost as a result of the adverse lifestyles.

	Population Attributable Risk (%)		Total avoidable lives lost
	Males	Females	
**All-cause**			
Drinking	0.162 (0.102, 0.222)	−0.018 (−0.070, 0.086)	680,466 (233,201, 1,297,515)
Smoking	0.186 (0.123, 0.239)	0.006 (0.004, 0.008)	867,530 (577,458, 1,120,579)
Diet	0.027 (−0.014, 0.073)	0.070 (0.040, 0.101)	352,537 (66,241, 664,837)
Inactivity	0.108 (0.076, 0.143)	0.055 (0.038, 0.074)	678,317 (470,584, 898,980)
Combined^a^	0.363 (0.244, 0.485)	0.108 (0.018, 0.227)	2,017,541 (1,174,247, 2,843,634)
**All-CVD**			
Drinking	0.077 (0.000, 0.156)	−0.037 (−0.103, 0.059)	82,492 (−153,791, 364,689)
Smoking	0.069 (−0.014, 0.148)	0.003 (0.001, 0.006)	126,553 (−21,961, 271,352)
Diet	0.005 (−0.053, 0.061)	0.050 (0.004, 0.095)	82,213 (−89,013, 248,478)
Inactivity	0.092 (0.051, 0.137)	0.063 (0.036, 0.093)	257,530 (143,305, 379,978)
Combined^a^	0.209 (−0.010, 0.368)	0.081 (−0.054, 0.216)	489,884 (−99,480, 973,212)
**All-cancer**			
Drinking	0.196 (0.131, 0.263)	−0.015 (−0.082, 0.062)	229,103 (105,554, 362,869)
Smoking	0.328 (0.266, 0.386)	0.009 (0.007, 0.011)	407,494 (329,118, 480,320)
Diet	0.014 (−0.038, 0.065)	0.050 (0.011, 0.092)	49,979 (−39,376, 140,449)
Inactivity	0.078 (0.041, 0.115)	0.038 (0.013, 0.061)	120,813 (58,376, 181,701)
Combined^a^	0.454 (0.341, 0.543)	0.079 (−0.047, 0.196)	607,517 (385,801, 794,445)

a The combined takes into account shared effects and is therefore not equivalent to the sum of the individual components.

## Discussion

In this large study of 33,411 individuals aged 30+ years each of the lifestyle factors, eating a poor diet, physical inactivity, excessive alcohol consumption or smoking were each associated with a higher odds of mortality with those having all four adverse lifestyles having a 285% higher odds of all-cause mortality relative to those with a healthy lifestyle. Modifying these lifestyle habits to the appropriate healthy form was estimated to reduce all-cause mortality by a quarter, which would prevent approximately 2 million deaths annually in China. There was a stronger effect of the adverse lifestyle factors on all-cancer mortality which could be reduced by almost a third, whereas all-CVD was reduced by 15% potentially saving 0.61 and 0.49 million lives, respectively.

The risk estimates from Western populations for all-cause, and where available, all-CVD and all-cancer, are similar in most of the analyses reported to date [7], [8], [9]. For instance, data from the UK Health and Lifestyle Survey investigating the same lifestyle risk factors as in the current study found similar levels of risk for all-cause mortality of 18%, 24%, 22% and 57% for diet represented by fruit and vegetable consumption, physical activity, alcohol intake and smoking, respectively [8]. The increase in risk associated with four adverse lifestyle factors relative to those with none, was also similar at 3.49 compared to 3.85 in the current study [8]. Given the differences in prevalence, the PAR% in the European studies tended to be higher at around 60% [7], [8].

Smoking was found to be the major preventable lifestyle determinant of mortality in most studies. In the current study this was also the case being associated with an excess of 0·87 million deaths. However, due to the low prevalence of smoking in Asian females [4], [14], [15], [16], [18], most of the deaths were in males, accounting for almost 20% of deaths. Although currently the impact of smoking is more limited in females, excluding the significant effect of passive smoking [14], [28], the situation is not static. Females are being actively targeted by tobacco manufacturers [29], and perceptions associated with smoking, such as being an indicator of social liberation are already present in teenage girls [30]. In South Korea, just one year after multinational tobacco companies were able to sell cigarettes in the country, the rate of smoking in teenage girls quintupled [31]. In the US Nurses’ Health Study smoking accounted for over one quarter of deaths in these females [11]. Were prevalence rates of active smoking to increase to those of males as is the case in Western populations, it was estimated there would be an additional 0.77 million deaths; and even if the prevalence rose to the more conservative levels observed in UK females (20%) [32], lives lost attributable to active smoking rose by 40%. These data clearly highlight the importance of preventing similar increases in smoking prevalence in the Chinese female population, and the need for reductions in the prevalence of male smoking to avoid the huge associated health burden.

However, the impact of the other lifestyle factors in China on the risk of mortality is less well known and the number of associated excess deaths has yet to be reported. The present study shows these habits are similarly important. Heavy alcohol drinking was associated with an excess of about 0.68 million, predominantly male deaths. Although some studies, but not all, suggest a benefit from moderate alcohol use [7], [10], particularly for CVD mortality, excessive alcohol intake is consistently associated with increased risk [8], [9], [11], including Chinese in other settings [17]. In males, Chinese alcohol consumption is integrally linked to social and business conventions and has been increasing along with related health and social problems, including domestic violence and traffic accidents [33], none of which are included in the current estimates. Of particular concern is, as with smoking, the high levels of consumption at a young age, particularly in females where annual consumption levels were similar to those in males (78% males vs 61% females) [33]. Legislation is being actively enforced to reduce traffic accidents. However, despite a number of high profile alcohol-related deaths [34], other areas of concern, such as the social implications, have been largely ignored. It is clear that action is warranted to reduce excessive alcohol intake in the male Chinese population.

Increasing mechanisation and computerisation in the workplace and at home, as well increased access to public and private transportation has resulted in marked reductions in physical activity [1]. The changes are in essence desirable reducing the burden of high-energy strenuous activities and are an indicator of improving social status. However, low levels of activity overall have negative health implications [1], [2], [3], as seen in the present study with 0.68 million excess deaths associated with physical inactivity, particularly for all-CVD mortality. In older Europeans, reduced physical activity was the strongest lifestyle determinant of mortality [7], other studies all show significantly increased risk although the relative importance varies [8], [9], [17].

Diet, as measured by daily fruit consumption was associated with the least number of excessive deaths, yet was still associated with an extra 0.35 million deaths. Similar associations have also been observed in Western populations [8], [9], [12] and in non-drinking, non-smoking, married Chinese women, [14] Chinese from Singapore [17], or Taiwanese diabetics [18]. Dietary changes from a predominantly vegetable and carbohydrate-based diet to one increasingly rich in animal protein and fat are rapidly occurring, although American-style fast food and soft drink consumption are not prevalent [1]. These dietary and physical activity changes have been linked to increased adiposity and the subsequent risk of both CVD, through concomitant increases in closely associated risk factors such as diabetes and hypertension, and cancer, which is reflected in the increased risk of death from these disorders in those with adverse dietary habits, particularly in the females.

The study has a number of strengths. It is the first to investigate all these lifestyle factors in a developing Asian population, using an ethnically homogeneous Chinese population. The study sample was large and included 81% of all deaths in Hong Kong. The risk estimates were adjusted for a range of potential confounding factors including age, sex, socioeconomic status (education, place of birth, housing, job type) and included mutual adjustment for the other lifestyle factors. The lifestyle factors were only crudely measured, which is inevitable given the very large sample size, the setting, and that information describing consumption 10 years earlier was used, but does reduce potential recall bias. The impact of the lifestyle factors might have been over-estimated if recall bias was present with under-reporting of intake in cases and/or over-reporting of intake in controls. This was, however, unlikely, since the effect of most lifestyle factors on mortality was not clear to most people and a wide range of lifestyle habits were investigated. The lifestyle data of the cases and controls were reported by the same proxy informant who were unaware of the research hypotheses to be tested, which would have further reduced the potential bias of self-reporting socially desirable information. It is also likely that Chinese societal communitarian values and a Confucian respect for authority may have further improved the accuracy of reporting, which is supported by the prevalence of the lifestyle factors in the present study being similar to that of a representative local survey [35], thus providing external validity. As these lifestyle factors are common and readily assessable, they are unlikely to be associated with any unmeasured major confounding factors that may explain the observed effects on mortality. As such the main issue is likely random error in reporting, particularly with older proxy reporters who may suffer from cognitive decline or memory loss, that would most likely attenuate any observed association, thus would be more of an issue ruling out a null result, which is not the case in the present study. However, remote recalling of lifestyle 10 years prior have been suggested to be as reliable as recent recall, suggesting this is less of an issue [36]. The use of crude measures of the lifestyle factors, although limiting issues with recall bias, may attenuate the impact on mortality. For instance, only incorporating fruit intake does not recognise the importance of known harmful food-related effects such as of trans-fats. Overall, this would attenuate the observed impact on diet on mortality. Thus the true effects are likely to be greater. Knowledge of the lifestyle factor exposure 10 y prior to mortality enables some potential inference of prior exposure and thus causality, however, confirmation using longitudinal data is required. Future studies may improve the mode fit by including more detail assessment on lifestyle behaviours.

### Conclusions

In summary, these adverse lifestyle habits were major determinants of mortality in the Chinese population with a 2 million excess deaths annually, and thus need to be targeted to reduce the health burden associated with the country’s rapid economic development. Smoking cessation and prevention of initiation, particularly in women, where levels are currently low are clearly important. However, the importance of the other lifestyle factors is less well recognised, yet heavy drinking and physical inactivity contribute to similarly large numbers of excess deaths annually. Initiatives are clearly required to increase physical activity levels, and to target excessive alcohol consumption, which is still promoted in the workplace as part of normal business practice. Targeting both smoking and alcohol will also have the advantage of reducing the bystander impact of passive smoking, spousal abuse and traffic accidents. Prevention is likely to be the only affordable approach to deal with the surge in cancer and CVD mortality.
